# RRAM-based parallel computing architecture using *k*-nearest neighbor classification for pattern recognition

**DOI:** 10.1038/srep45233

**Published:** 2017-03-24

**Authors:** Yuning Jiang, Jinfeng Kang, Xinan Wang

**Affiliations:** 1 Institute of Microelectronics, Peking University, Beijing 100871, China; 2 The Key Laboratory of Integrated Microsystems, Peking University Shenzhen Graduate School, Shenzhen 518055, China

## Abstract

Resistive switching memory (RRAM) is considered as one of the most promising devices for parallel computing solutions that may overcome the von Neumann bottleneck of today’s electronic systems. However, the existing RRAM-based parallel computing architectures suffer from practical problems such as device variations and extra computing circuits. In this work, we propose a novel parallel computing architecture for pattern recognition by implementing *k*-nearest neighbor classification on metal-oxide RRAM crossbar arrays. Metal-oxide RRAM with gradual RESET behaviors is chosen as both the storage and computing components. The proposed architecture is tested by the MNIST database. High speed (~100 ns per example) and high recognition accuracy (97.05%) are obtained. The influence of several non-ideal device properties is also discussed, and it turns out that the proposed architecture shows great tolerance to device variations. This work paves a new way to achieve RRAM-based parallel computing hardware systems with high performance.

Although artificial neural networks (ANNs)^1^,^2^,^3^ and many other machine learning (ML)^4^,^5^,^6^ algorithms implemented on von Neumann computers have achieved great success in pattern recognition tasks, the limitations of sequential von Neumann architecture are exposed when facing extremely massive data and calculation. The limited access speed^7^,^8^,^9^,^10^ has become the major performance bottleneck of sofware-based pattern recognition systems. Hardware-realized parallel computing architectures are feasible solutions to this problem. For example, many RRAM-based neuromorphic systems with massive parallelism have been proposed in the past few years^11^,^12^,^13^,^14^. The simple structure, remarkable scalability, fast switching speed and low operating power have made RRAM a promising device for parallel computing implementations^12^,^15^,^16^,^17^. The tunable resistance of RRAM has made it possible to realize both storage and computing in the same device. Now that RRAM has been used as a crucial device of neuromorphic computing architectures, there are still some practical problems. Training is the major difficulty of such architectures due to the complexity of neural network algorithms. When training a neuromorphic system, a learning rule must be implemented, such as the perceptron training rule, BP^18^, STDP^19^, and Manhattan update rule^13^. If example attributes are used as synaptic weights directly^14^, the neuromorphic system can not handle the learning of multiple examples with the same label well. In other words, neuromorphic systems always require massive computing to determine their synaptic weights, which makes them still dependent on von Neumann architecture, or results in extra hardware overhead. In addition, device variations may significantly reduce the recognition accuracy of neuromorphic systems^20^.

A novel computing architecture that implements *k*-nearest neighbor (*k*NN) on RRAM arrays can get rid of these problems. *k*NN^21^,^22^,^23^, which is another machine learning method that has made enormous achievements in pattern recognition, hasn’t been implemented on advanced RRAM devices yet in the previous researches. In this paper, we present a metal-oxide RRAM-based parallel computing architecture that uses *k*NN classification for pattern recognition. The proposed architecture is also tested by the Modified National Institute of Standards and Technology (MNIST) handwritten digit database^1^. It turns out that the proposed architecture achieves high speed and high recognition rate, and shows great tolerance to device variations. In addition, the training of the proposed architecture is simple with no need for weight computing circuits. For the first time, the implementation of *k*NN algorithm on RRAM arrays is proposed. Our results suggest a new research direction for hardware-realized pattern recognition systems with high performance.

## Results

### Description of the proposed architecture

**Figure 1:**
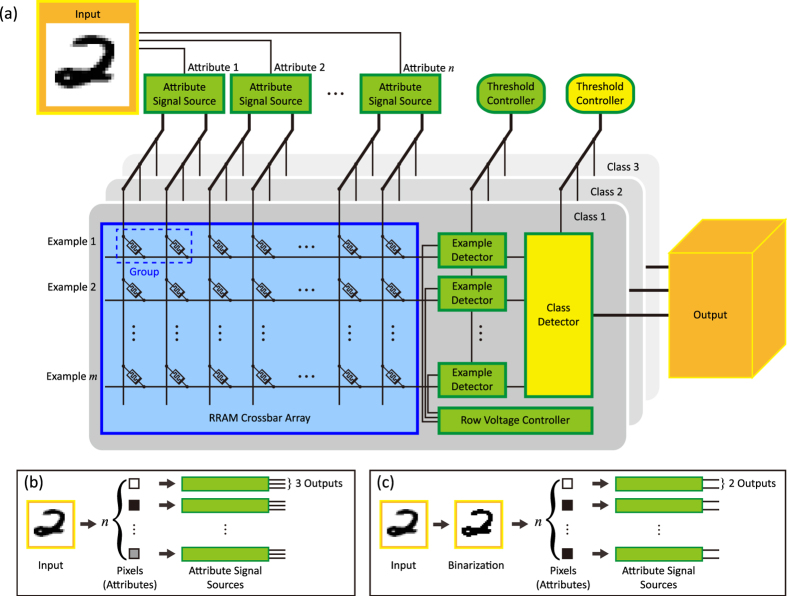
Overview of the proposed architecture. (**a**) The proposed RRAM-based parallel computing architecture for pattern recognition. (**b**) Signal conversion for the multilevel implementation. (**c**) Signal conversion for the binary implementation.

As shown in Fig. 1(a), the proposed architecture consists of attribute signal sources, RRAM crossbar arrays, example detectors, class detectors, threshold controllers and row voltage controllers. Attribute signal sources generate voltage signals according to the attribute values they accept, and apply the signals to the columns they connect to. RRAM crossbar arrays are the center of storage and computing. TiN/HfO_*x*_/AlO_*x*_/Pt RRAM arrays with remarkable characteristics are used in this work (see Supplementary Figs S1–3). Note that there are several arrays with identical size in the proposed architecture. During the training process, examples with the same label are stored in the same array. During the classifying process, RRAM arrays computes the outputs of a similarity function in parallel. An example detector with an operational amplifier and a comparator detects whether the relative stored example is close enough to the input example. Each example detector is responsible for one row of the RRAM array. Class detectors are quite similar to example detectors, except that they detect whether the relative class is close enough to the input example. Each class detector is responsible for one RRAM array. There are two threshold voltage controllers, which controls the threshold voltage of example detectors and class detectors, respectively. The row voltage controllers determine the voltage applied to the bottom electrodes of RRAM cells in each row, and in this way, they control the read/write of the array.

For different types of input data, the concrete implementations are a bit different. The multilevel implementation is proposed for real-valued input data, while the binary implementation is proposed for binary input data. These two types of implementations are different in signal conversion, RRAM arrays and array operations, but uniform in the rest parts. The schematic diagrams of signal conversion for multilevel implementation and binary implementation are shown in Fig. 1(b) and (c), respectively. Each attribute signal source processes one pixel of the input image, which is defined as one attribute of an example. Each input pattern contains *n* pixels with grey levels, in other words, there are *n* attributes in each example. Therefore, *n* attribute signal sources form the interface between the input pattern and the RRAM crossbar arrays. Attribute signal sources of multilevel implementation accept grey level values directly, and each of them provides three outputs. Attribute signal sources of binary implementation accept binary values, and each of them provides two outputs. Details are discussed in the following sections.

The proposed architecture operates in two different modes: training and classifying. The proposed architecture is trainable, and its training process can be completely independent of von Neumann computers. In the training process, example data is written into the arrays. After training, RRAM crossbar arrays actually store all informations of all the trained examples. In the classifying process, the proposed architecture looks up the memory and finds out the class that is the closest to the input example according to the principle of *k*NN algorithm. The particularity of this architecture is that no complex computing for learning rules is required in the training process, and the detection of similarity between the input example and each of the trained examples in the classifying process is parallel. In this way, high efficiency is achieved.

### Implementation of *k*NN classification

In machine learning, *k*NN is known as a lazy learner^21^. In contrast to learning methods (i.e. neural networks) that construct a general, explicit description of the target function when training examples are provided, *k*NN simply stores the training examples rather than performs explicit generalization first. Generalization beyond these examples is postponed until a new example must be classified^24^.

The proposed architecture can perform *k*NN classification in parallel by using the nature of RRAM crossbar arrays. We already know that matrix-vector multiplication (or weighted sum) can be easily achieved on RRAM crossbar arrays^20^, thus, these arrays can be used as synaptic weights of neuromorphic systems. Now we find that the same crossbar architecture can compute squared Euclidean distance as well, which is the most crucial variable in *k*NN algorithm.

Suppose that the architecture has been trained by a sequence of examples 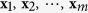, and then a newly input example **x** is given. An arbitrary example **x**_*i*_ can be described as the following feature vector in equation (1):


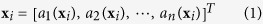


Where *a*_*j*_(**x**_*i*_) denotes the value of the *j* th attribute of example **x**_*i*_.

Thus, the squared Euclidean distance between the newly input example **x** and a trained example **x**_*i*_ can be defined by equation (2):


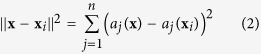


If equation (2) is transformed properly, it can be worked out by a parallel computing architecture based on RRAM crossbar arrays.

If *a*_*j*_(**x**) and *a*_*j*_(**x**_*i*_) contain real number, we should employ the multilevel implementation. First of all, equation (2) is expanded by the binomial theorem, as shown in equation (3).


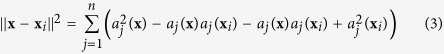


It’s worth noting that the coefficient of each term is 1, since normalized voltage and conductance are used. Express each term in equation (3) as voltage and conductance, we get equation (4):


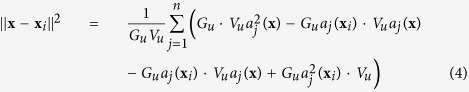


**Figure 2:**
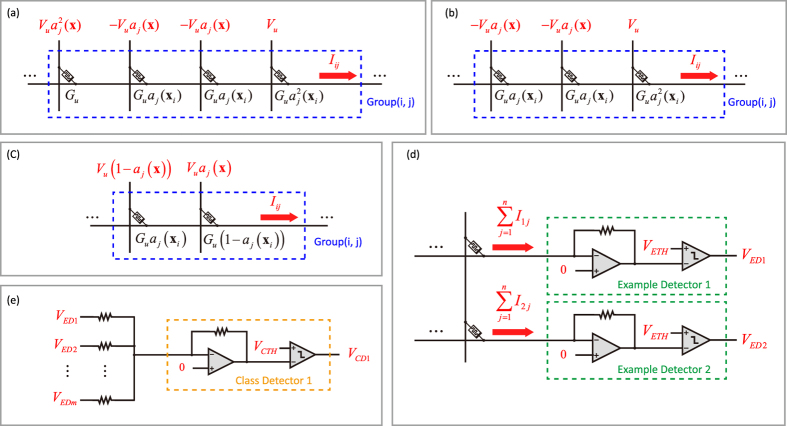
Schematic view of the implementation of *k*NN classification on the proposed architecture. (**a**) Array calculation of the multilevel implementation. (**b**) Simplified array calculation of the multilevel implementation. (**c**) Array calculation of the binary implementation. (**d**) Schematic view of example detection. (**e**) Schematic view of class detection.

Where *G*_*u*_ is the unit conductance of RRAM, and *V*_*u*_ is the unit read voltage. The hardware implementation of equation (4) is shown in Fig. 2(a). In the multilevel implementation, four RRAM cells form one group together. If each example possesses *n* attributes, there are *n* groups in each row. Voltages are applied to each column, and the output current of a row implies the result of the squared Euclidean distance between a trained example and the newly input example. Note that the second term and the third term in equation (4) is identical, yet we still require two cells to do the calculation respectively, in order to decrease the influence of device nonlinearity. For a given input example, the first term in equation (4) is irrelevant to the attributes of any trained examples. When the squared Euclidean distance compares with each other, the first term is a constant and has no effect on the result. Therefore, the first term can be omitted, and the design of a group in the multilevel implementation can be simplified as Fig. 2(b).

For the binary implementation, the design can be further simplified. By using the laws of Boolean algebra, equation (3) can be rewritten as equation (5):


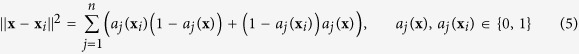


Express each term in equation (5) as voltage and conductance, we get equation (6):


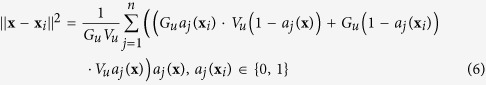


The hardware implementation of equation (6) is shown in Fig. 2(c).

Therefore, the RRAM crossbar arrays allow highly parallel computing of squared Euclidean distance. Now that squared Euclidean distance is obtained, the architecture needs to search for the trained examples in the neighborhood of the input example. In other words, the architecture should compare all the squared Euclidean distances and find out the closest class, and then, the input example is classified as this class. To achieve this, a dynamic threshold method is used in this work.

During classification, the proposed architecture requires two operation phases: example detection and class detection. It is obvious that squared Euclidean distance is used as a measure of example similarity. The smaller the squared Euclidean distance is, the more similar the two examples are. In the example detection, the architecture obtains the squared Euclidean distance between the input example and each trained example, and judges whether they are close enough. If so, the example detector generates a high-level output. Circuits for example detection is shown in Fig. 2(d). Each example detector consists of a feedback resistor, an operational amplifier and a comparator. The operational amplifier outputs a static voltage that represents the result of squared Euclidean distance. Then a dynamically varying threshold, *V*_*ETH*_, is applied. *V*_*ETH*_ is generated by a threshold controller, and all the example detectors in the architecture share the same threshold voltage. *V*_*ETH*_ rises from the lowest voltage. When *V*_*ETH*_ is larger than the output of an amplifier, the corresponding example detector generates a high-level output, which implies that the trained example is close enough to the input example. When a certain amount of example detectors turn high, *V*_*ETH*_ stops rising and keep its value, so that all the example detectors keep their output. At this time, class detection begins. The schematic view of class detection is shown in Fig. 2(e). The operation of class detection is similar to that of example detection, expect that class detection compares the number of example detectors with high-level outputs, and search for the closest class. There is a global threshold voltage as well, namely, *V*_*CTH*_. *V*_*CTH*_ rises from the lowest voltage. When *V*_*CTH*_ reaches the output voltage of one of the amplifiers, it stops rising and keeps its value. Now there must be only one class detector gives a high-level output, and it represents the closest class as well as the final result.

### Training operations

The proposed architecture is trainable. During the training process, we write attribute informations of all the training examples into the arrays without extra weight computing. The cost of training is low.

**Figure 3:**
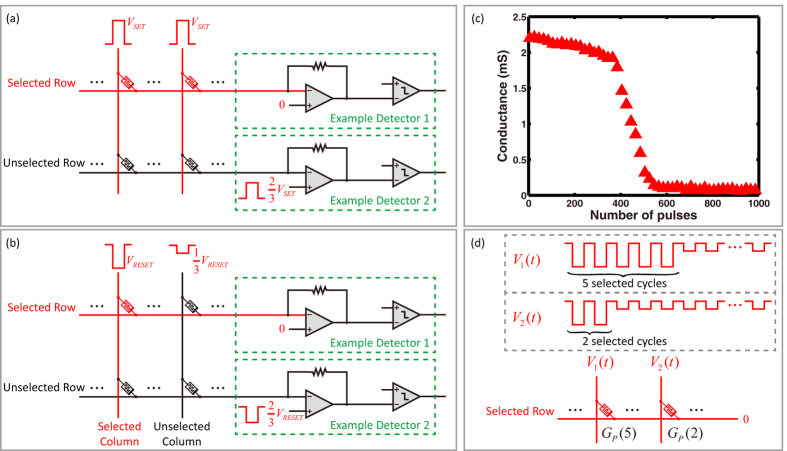
Training method for the proposed architecture. (**a**) SET operations in the training process. (**b**) RESET operations in the training process. (**c**) Measured RRAM conductance as a function of number of applied negative pulses. (**d**) Training scheme of the multilevel implementation.

Transistor-free operation has already been experimentally proved to be feasible^13^. To avoid unintended switching of RRAM cells, a 1/3 bias scheme is employed^25^. The training of the architecture includes SET operations and RESET operations (see Supplementary Figs S4–5 and Supplementary Table S1). Training pulses with different amplitude are simultaneously applied to the top electrodes and bottom electrodes of RRAM cells. The pulses for top electrodes are generated by attribute signal sources and directly applied to each column, yet the pulses for bottom electrodes are generated by row voltage controllers and applied to the non-inverting input of the amplifiers. By using the feedback of amplifiers, pulses are applied to bottom eletrodes indirectly. The SET operation is illustrated in Fig. 3(a). When the training starts, only one row is selected at a time. In the SET operation, all cells in the selected row are SET to the low resistance state, which implies that all data stored in the row is deleted. The RESET operation is illustrated in Fig. 3(b). RESET operations write data into the memory. Only one row and several columns are selected. Only the selected cells are modified. For the binary implementation, the selected cells are directly RESET to the high resistance state, and only one RESET pulse is needed. For the multilevel implementation, we need to modify the conductance of selected cells gradually. Gradual conductance change of the RRAM device is required. By controlling the number of applied negative pulses, gradual conductance change is obtained in our RRAM devices, as shown in Fig. 3(c). Therefore, a training scheme of the multilevel implementation is proposed in Fig. 3(d). For RRAM cells to be modified to different conductance, we employ different number of selected cycles. During the selected cycles, we apply negative pulses with full amplitude to the selected columns. While during the unselected cycles, we apply negative pulses with 1/3 amplitude to the selected columns. The more selected cycles we employ, the lower conductance the cell will change to. *G*_*P*_(*x*) is the intended conductance as a function of number of selected cycles.

### MNIST testing and performance analysis

**Figure 4:**
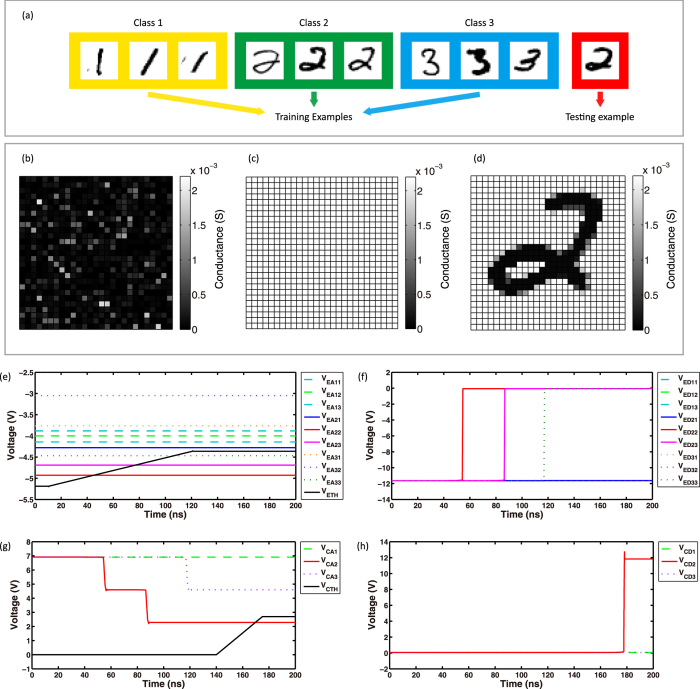
SPICE simulation results of the proposed architecture. (**a**) Training examples and testing examples used in the SPICE simulations. (**b**) Initial conductance of RRAM cells. (**c**) Conductance of RRAM cells after the SET process. (**d**) Conductance of RRAM cells after the RESET process. (**e**) Outputs of amplifiers in example detectors and the dynamic threshold for example detectors during the classifying process. (**f**) Outputs of example detectors during the classifying process. (**g**) Outputs of amplifiers in class detectors and the dynamic threshold for class detectors during the classifying process. (**h**) Outputs of class detectors during the classifying process.

We have verified and evaluated the proposed computing architecture in simulations. The architecture is tested by the MNIST database, which includes a training set of 60,000 examples and a test set of 10,000 examples. Figure 4(a) shows several examples from the MNIST database. We use the nine training examples and one testing example for our SPICE simulation. Figure 4(b,d) illustrate the simulation results of training for the multilevel implementation. At the beginning, all the RRAM cells are initialized by random conductance from 50 μS to 2 mS. Then read pulses with 0.1 V amplitude are used to measure the conductance of RRAM cells, the results are shown in Fig. 4(b). Then the SET process is performed, all the RRAM cells switch to the low resistance state. After the SET process, the conductacne of RRAM cells is read again, the results are shown in Fig. 4(c). Then the RESET process is performed, attribute informations of an example is written into the arrays. Finally we obtain Fig. 4(d), which proves the training method is successful.

Figure 4(e,h) are the simulated waveforms during the classifying process. An untrained handwritten digit “2” (Fig. 4(a)) is given to the circuits. In the simulation we define *k* = 3, therefore, as soon as the dynamic threshold for example detectors is higher than three amplifier outputs, it stops varying. Every time when the threshold passes an amplifier output, an example detector switches to high-level. Things are similar in the class detectors. In Fig. 4(h), we can see that the class detector that represents class “2” gives a high-level output, in other words, the newly input example is classified as “2”. Furthermore, the proposed architecture is extremely fast, the classification of each example only takes about 200 ns. In conclusion, the classifying process is successful.

**Figure 5:**
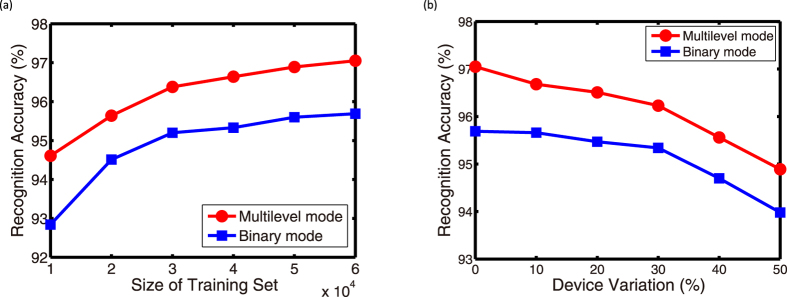
Recognition accuracy simulation results by applying the MNIST database. (**a**) Recognition accuracy as a function of the size of the training set. When all the 60,000 instances are learnt, the proposed architecture achieves a recognition accuracy of 97.05% for multilevel mode and 95.69% for binary mode. (**b**) Recognition accuracy as a function of the device variation. Great tolerance to device variations is shown by the proposed architecture.

To further verify the proposed architecture, we also apply the whole MNIST database to simulations. As shown in Fig. 5(a), the more examples the architecture has learnt, the higher recognition accuracy it achieves. In ideal conditions, when all the 60,000 examples are learnt, the architecture achieves a recognition accuracy of 97.05% for the multilevel implementation and 95.69% for the binary implementation. These results are better than that of existing neuromorphic systems^19^,^20^. Then the influence of several non-ideal device properties is evaluated. In this paper, device variation is defined as the ratio of the standard deviation of device resistance and mean resistance. All the 60,000 training examples are learned by the architecture, and then the 10,000 unlearned examples are used to test it. We find that the proposed architecture shows great tolerance to device variations, as shown in Fig. 5(b). The recognition accuracy does decrease with the increase of device variations, but even when the device variation is as high as 50%, the recognition accuracy is still sustainable (94.89% for the multilevel implementation and 93.98% for the binary implementation). The great tolerance to device variations can be explained by the learning ability of the architecture towards multiple examples. When the array is trained by large amount of similar examples, the variations of devices are averaged out. Device variations are the major issue of the current RRAM devices, and the proposed architecture is likely to overcome this issue. In addition, we also test the performance of the proposed architecture under limited ON/OFF ratio of RRAM devices. Interestingly, the recognition accuracy seems to be irrelevant to the ON/OFF ratio of RRAM devices due to the nature of the algorithm (see Supplementary Fig. S6).

## Discussion

For the first time, the implementation of *k*NN algorithm on RRAM arrays is proposed. Taking advantage of Ohm’s law and Kirchhoff ’s Current Law (KCL), the architecture computes squared Euclidean distance in parallel, which is the most computationally intensive part of *k*NN algorithm. Therefore, massive parallelism is realized. It is widely known that the major issue of software-realized *k*NN algorithm is the massive computation required to classify a new example^1^,^21^. When facing numerous examples, the recognition time is extremely large. Yet in the proposed parallel computing architecture, this issue no longer exists. Metal oxide RRAM, which is a novel nonvolatile memory, is used as both the storage and computing component. It stores data and provides strong computation power as well. In this architecture, the recognition time may not be influenced by the data size. Thus, the proposed architecture is competitive when facing massive data. And to some extent, this type of computing architecture is a promising alternative to overcome the limitations of von Neumann architecture.

On the other hand, *k*NN is particularly suitable for RRAM devices. In comparison with many neuromorphic systems, *k*NN requires fewer peripheral circuits, almost no computing is needed during training. Based on *k*NN, concrete hardware implementation is proposed for pattern recognition. With no extra computing circuits for training, the architecture is cheaper, and easier to be implemented on hardware. The proposed architecture takes advantages of the parallel computing ability of crossbar RRAM arrays, and peresents good performance in recognition. High recognition accuracy and high recognition speed is obtained. The architecture shows great tolerance to some non-ideal device properties like device variations. In addition, the SPICE simulation results show that the proposed architecture is robust and viable.

The use of RRAM may greatly optimize the speed and area of the pattern recognition systems. This work provides theoretical guidance and reference for the further implementation of high-performance pattern recognition hardware systems and more promising metal-oxide RRAM based applications.

## Methods

### Device structure and fabrication

The RRAM arrays mentioned in this work contain TiN/HfO_*x*_/AlO_*x*_/Pt cells. Each RRAM cell consists of a TiN top electrode, a Pt bottom electrode, and HfO_*x*_/AlO_*x*_ resistive switching layers sandwiched by them (see Supplementary Fig. S1). Firstly, on a SiO_2_/Si substrate, a Ti adhesion layer and a Pt bottom electrode layer (Ti/Pt 200 Å/1000 Å) are deposited by physical vapor deposition (PVD). Then patterning is performed by photolithography and lift-off. After fabricating the bottom electrodes, the growth of a SiO_2_ isolation layer (200 Å) is performed by plasma-enhanced chemical vapor deposition (PECVD). On the isolation layer, via holes are etched. After that, HfO_*x*_/AlO_*x*_ resistive switching layers are deposited successively by ALD (Picosun, Masala, Finland) at 300 °C, using trimethylaluminum (TMA)/tetrakis[ethylmethylamino]hafnium (TEMAH) and H_2_O as precursors, respectively. This process is repeated for 10 times, and the total thickness of HfO_*x*_ and AlO_*x*_ layers are 30 Å and 20 Å, respectively. Finally, a TiN layer (400 Å) for top electordes is sputtered, and patterned by photolithography and dry etching. Except the different parameters in the ALD process for resistive switching layers, the rest details of the fabrication process have been reported in our previous work^26^.

### Experimental measurement

The RRAM devices have been tested under the DC mode and the pulse mode on Agilent instruments. In the measurement, the bottom electrode of the device is grounded, and then voltage pulses are applied to the top electrode. An Agilent B1500A semiconductor device analyzer is used. In the DC mode, a ramped voltage is applied to the top electrode. The voltage gradually increases from 0 V to 2 V, then gradually decreases to 0 V, and then to −2.5 V, finally back to 0 V. In the pulse mode, the device is SET to the low resistance state by a positive pulse (1.8 V, 100 ns). Then a read pulse with a 0.1 V amplitude is applied to read the conductance of the device. And then, RESET pulses (−1.3 V, 100 ns) and read pulses are applied alternately. Both in the DC mode and the pulse mode, abrupt SET and gradual RESET characteristics can be obtained (see Supplementary Figs S2–3).

## Additional Information

**How to cite this article**: Jiang, Y. *et al*. RRAM-based parallel computing architecture using *k*-nearest neighbor classification for pattern recognition. *Sci. Rep.* **7**, 45233; doi: 10.1038/srep45233 (2017).

**Publisher's note:** Springer Nature remains neutral with regard to jurisdictional claims in published maps and institutional affiliations.

## Supplementary Material

Supplementary Information
