# Supplementary material for: RRAM-based parallel computing architecture using k-nearest neighbor classification for pattern recognition
Source: Sci Rep. 2017 Mar 24;7:45233. doi: 10.1038/srep45233 (PMC5364413; doi:10.1038/srep45233)
Supplement: Supplementary Information [file srep45233-s1.pdf]

Supplementary Information

RRAM-based parallel computing architecture using *k*-nearest neighbor classification for pattern recognition

Yuning Jiang[1†], Jinfeng Kang[1†] and Xinan Wang[2†]

[1]Institute of Microelectronics, Peking University, Beijing 100871, China
[2]The Key Laboratory of Integrated Microsystems, Peking University Shenzhen Graduate School, Shenzhen 518055, China

† Corresponding: Yuning Jiang (email: jiangyuning@pku.edu.cn) or Jinfeng Kang (email: kang_jf@163.com) or Xinan Wang (email: wang_xa@163.com).

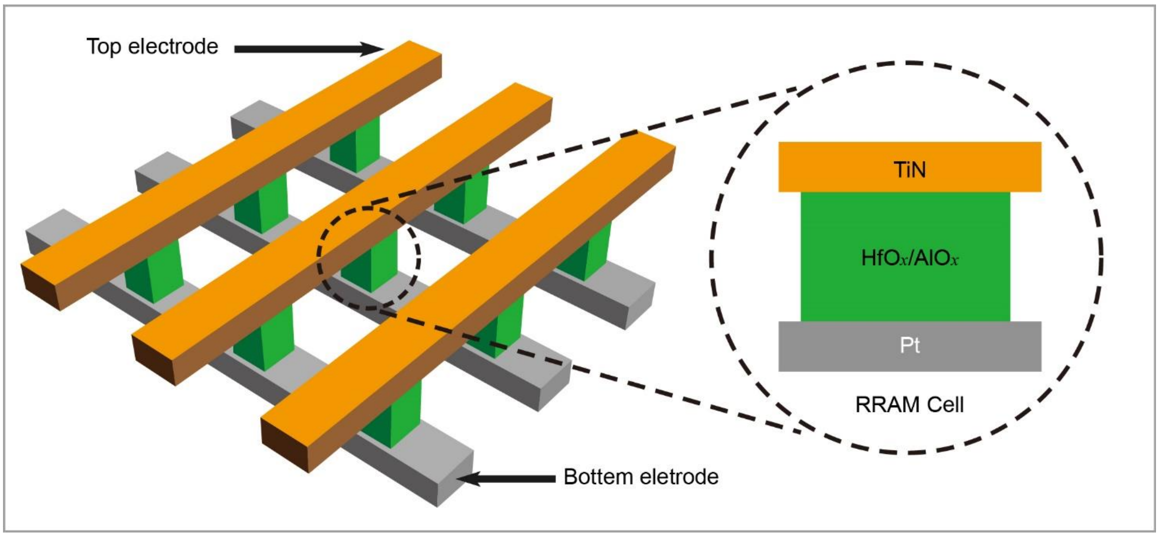

Supplementary Figure S1 | Schematic view of the transistor-free crossbar structure of RRAM arrays. RRAM cells are formed in each cross point of the array. TiN is used as the top electrode, Pt is used as the bottom electrode, and HfO_x/AlO_x layers are sandwiched in the middle as functional layers. TiN/HfO_x/AlO_x/Pt RRAM arrays with transistor-free structure are investigated in this work. Transistor-free crossbar, also known as 0T1R structure, means that the arrays are without any selection devices. Transistor-free crossbar RRAM arrays can exhibit extremely high-density integration ability ($4F^2$)[1-3], and they are much easier to scale than those with an additional transistor at each cross point[1,4]. Transistor-free operation has already been experimentally proved to be feasible[5]. Although the transistor-free crossbar structure enables the arrays to achieve extremely high density, there are some problems. As a matter of fact, the fixed structure greatly restrains the design of algorithms. In the circuit designing, we can not access a specific RRAM cell arbitrarily. Only several simple versions of artificial neural networks can be achieved before. But now in this work, *k*NN algorithm is available as well. The transistor-free crossbar structure also suffers from substantial sneak path leakages, therefore, the training of the array can be difficult. That's the reason why we employ the 1/3 bias scheme in this work. Our RRAM arrays contain TiN/HfO_x/AlO_x/Pt cells. Among the metal oxide materials, HfO_x-based devices have shown excellent performance such as fast switching speed, excellent switching endurance, and reliable data retention[6]. In other work[7], 4Mbit RRAM with HfO_x has demonstrated a very fast, 7.2ns read and write. Details of the fabrication process of the RRAM arrays used in this work have been reported in our previous work[8].

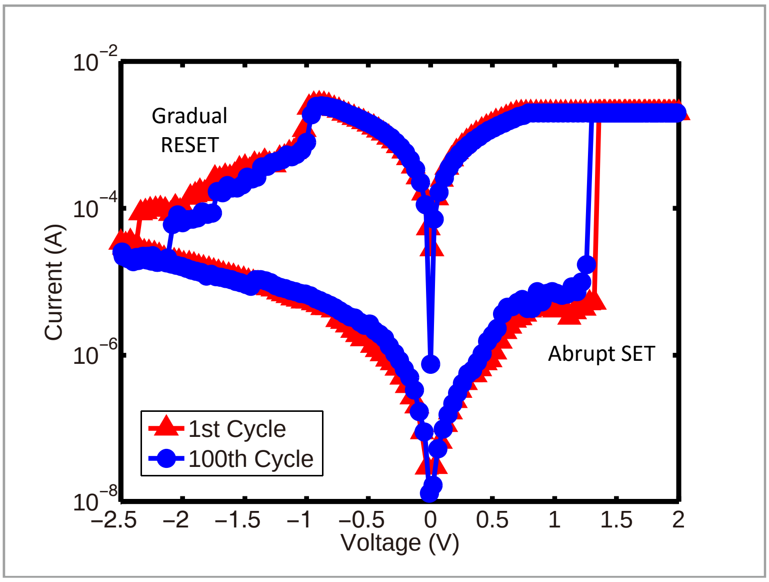

Supplementary Figure S2 | Electrical characteristics of the TiN/HfO_x/AlO_x/Pt RRAM in DC mode.

I-V characteristics of TiN/HfO_x/AlO_x/Pt RRAM are measured by a DC double sweep. An Agilent B1500A is used in the measurement. The bottom electrode of the device is grounded, and then a ramped voltage is applied to the top electrode. It's worth noting that our RRAM devices possess asymmetric resistive switching characteristics, which include an abrupt SET and a gradual RESET. During the SET process, as soon as a certain threshold voltage is reached, the RRAM switches from its high-resistance state (HRS) to low-resistance state (LRS), During the RESET process, it switches from LRS to HRS. During SET, a 2mA current compliance was enforced for the prevention of permanent breakdown of the device. It is widely accepted that a large nonlinearity in the I-V characteristics of the metal-oxide RRAM itself is needed to mitigate the leakage current of the array[9], and our RRAM meets this requirement well. In addition, when the applied voltage is as low as 0.1V, nondestructive read can be achieved. The DC double sweep measurement has been repeated for 100 times, and it turns out that the characteristics of RRAM are relatively stable.

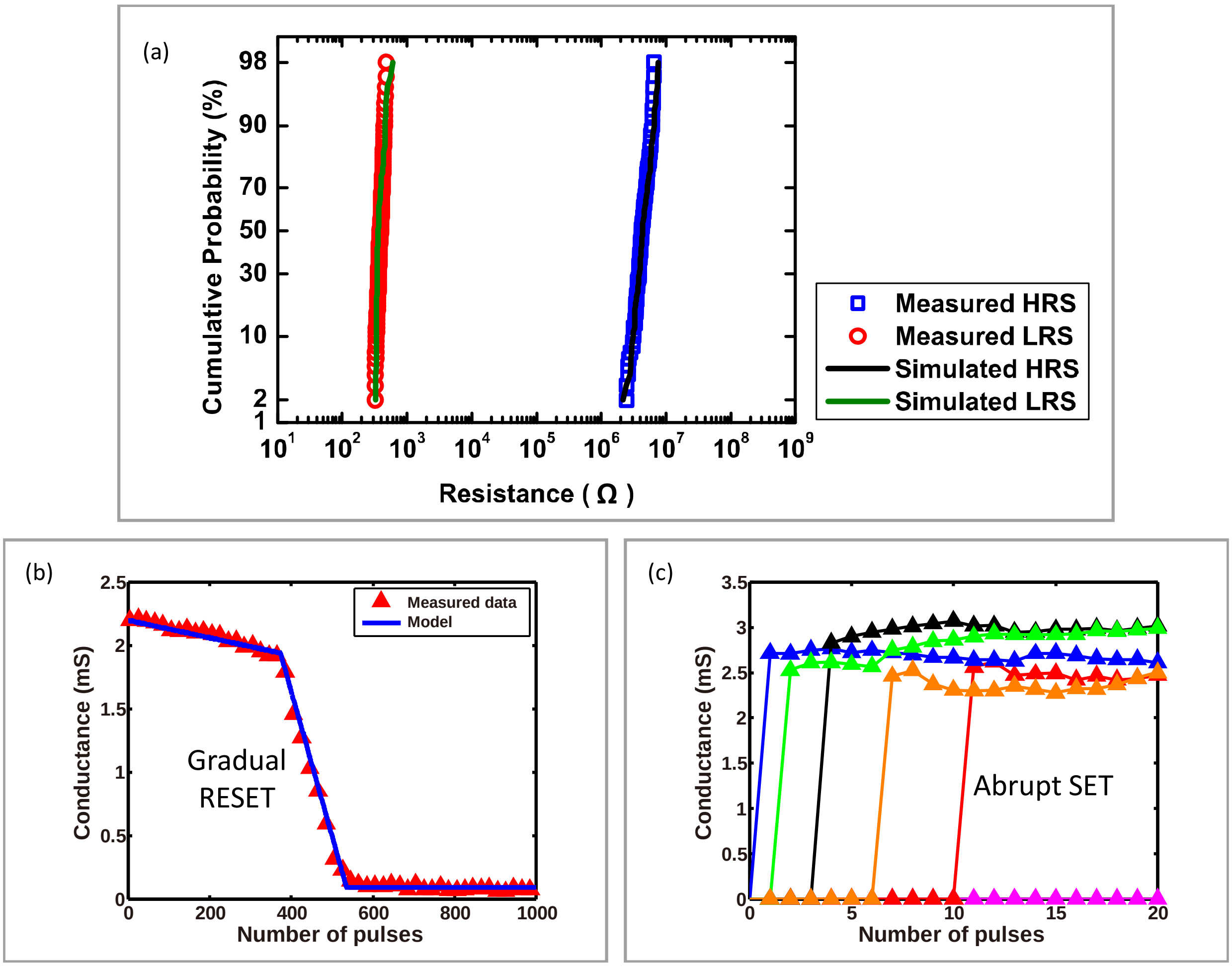

Supplementary Figure S3 | Electrical characteristics of the TiN/HfO$_x$/AlO$_x$/Pt RRAM in pulse mode. **(a)** Measured and simulated distributions of LRS and HRS states in pulse mode. When the applied SET voltage pulses (1.15V, 100ns) and RESET voltages pulses (-1.60V, 100ns) are large enough, the RRAM device can switch between two stable resistance states. A resistive switching operation only takes one pulse. **(b)** Gradual RESET characteristics in pulse mode. When the RESET voltage pulses are of a relatively small critical amplitude and a suitable pulse width (-1.30V, 100ns), the device shows gradual RESET characteristics. **(c)** Stochastic SET characteristics in pulse mode. Unlike the behaviors in a RESET process, even if the amplitude of the SET voltage pulses is small enough, the device does not show gradual characteristics. When the SET voltage pulses are of a critical amplitude and a suitable pulse width (1.00V, 100ns), the device shows stochastic SET characteristics, which are still abrupt. Such stochastic SET characteristics have been reported before[10]. Yet in this work, we only use the deterministic abrupt SET characteristics. In all these experiments, an Agilent B1500A semiconductor device analyzer, an Agilent 34980A multifunction switch/measure unit, and an Agilent 81160A pulse function generator are used.

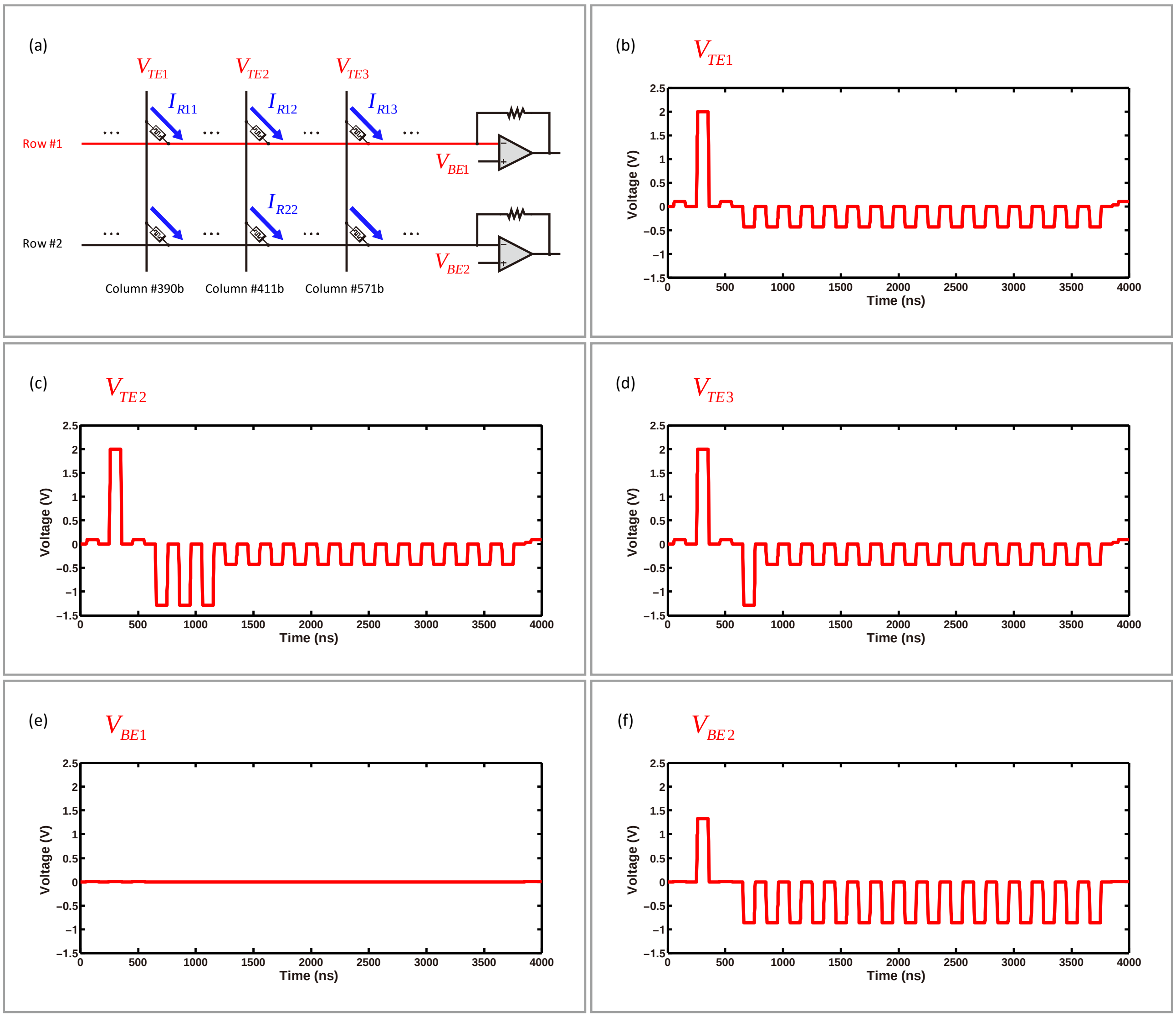

Supplementary Figure S4 | Partial RRAM cells and operation voltage waveforms in the SPICE simulation. **(a)** Examples of the array operation in multilevel mode. Voltages for top electrodes are applied directly to each column, while voltages for bottom electrodes are applied to the non-inverting input of the amplifiers. Row #1 is selected. All the RRAM cells in the unselected Row #2 should keep their conductance unchanged. SPICE simulations have been performed to verify the operation of the training process. Now several RRAM cells in the SPICE simulation of the multilevel implmentation are chosen as examples to illustrate the details. Three columns are chosen: Column #390b, Column #411b and Column #571b. **(b)** The top electrode voltage of Column #390b. At about t = 100ns, a read pulse is applied. At about t = 300ns, the SET process is conducted. At about t = 500ns, another read pulse is applied. Then by employing different number of "strong" pulses (V_{RESET}) and "weak" pulses (1/3 V_{RESET}), the RESET process is conducted. Finally at t = 4000ns, we read once again. **(c)** The top electrode voltage of Column #411b. **(d)** The top electrode voltage of Column #571b. **(e)** The bottom electrode voltage of the selected Row #1. The bottom electrode voltage of the selected Row #1 is always 0V. **(f)** The bottom electrode voltage of the unselected Row #2. The bottom electrode voltage of the unselected Row #2 is always 2/3 of the operational voltage, i.e., V_{SET} or V_{RESET}.

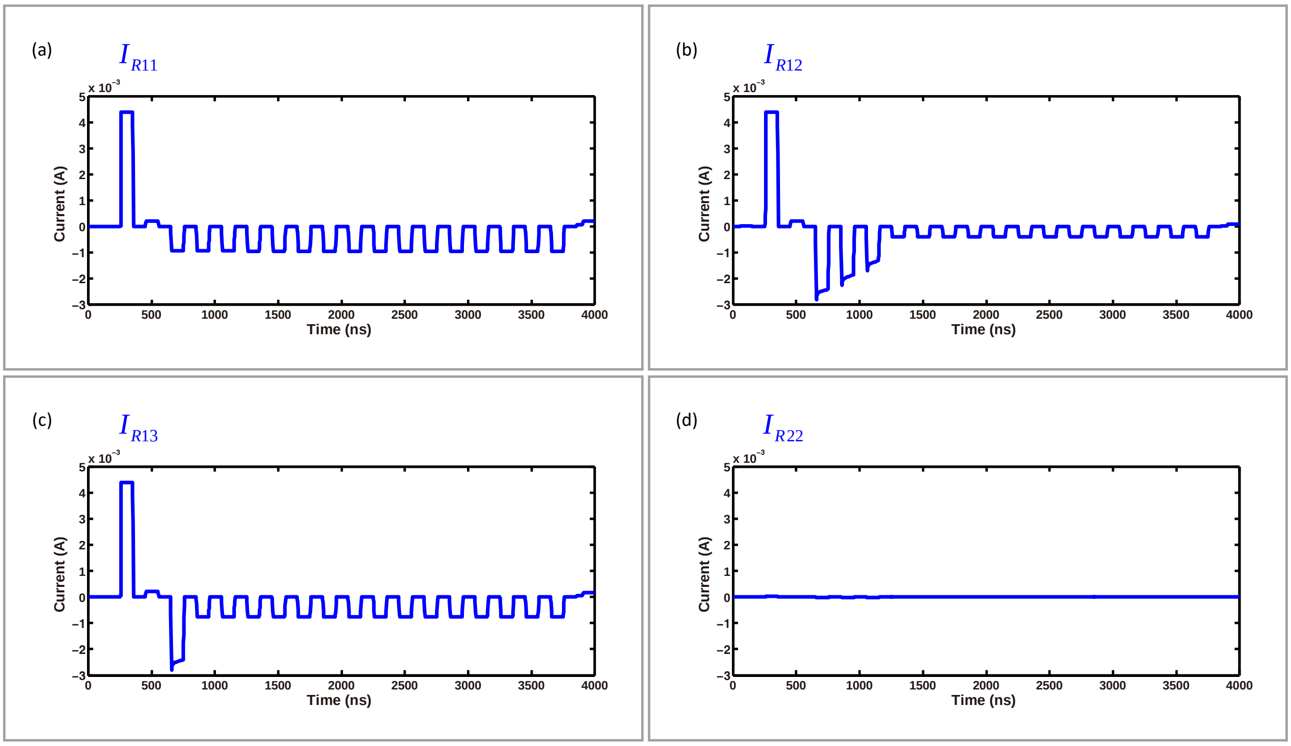

Supplementary Figure S5 | Currents through the RRAM cells in the SPICE simulation.

(a) The current through the RRAM cell in Column #390b, Row #1. **(b)** The current through the RRAM cell in Column #411b, Row #1. **(c)** The current through the RRAM cell in Column #571b, Row #1. **(d)** The current through the RRAM cell in Column #411b, Row #2.

Supplementary Table S1

The conductance of RRAM cells in different period of the simulation (Unit: S)

	t = 100ns	t = 500ns	t = 4000ns
(#1, #390b)	2.19×10^{-5}	2.20×10^{-3}	2.20×10^{-3}
(#1, #411b)	1.25×10^{-4}	2.20×10^{-3}	9.38×10^{-4}
(#1, #571b)	4.48×10^{-5}	2.20×10^{-3}	1.80×10^{-3}
(#2, #411b)	2.93×10^{-5}	2.93×10^{-5}	2.93×10^{-5}

The conductance of the RRAM cells discussed above in different period is shown in this table. It can be observed that the conductance changes of each cell is different. At first, the RRAM cells are initialized with random conductance. And after the SET process, all cells in the selected Row #1 switch to the low resistance state, while the RRAM cell in the unselected Row #2 does not change its conductance. After the RESET process, in Row #1, three different conductance values are obtained, while in Row #2, the conductance remains unchanged.

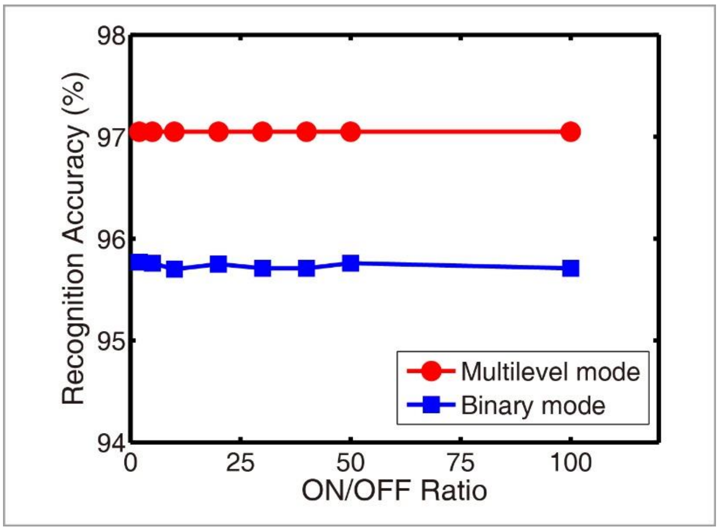

Supplementary Figure S6 | Recognition accuracy as a function of the ON/OFF ratio in the MNIST simulations.

Another discovery is the ON/OFF ratio immunity. 60,000 training examples and 10,000 testing examples are used in the MNIST simulations. Suppose that the RRAM cells have limited ON/OFF ratio, then RRAM cells are configured with proper conductance in the limited range. As the result implies, the proposed architecture seems to be immune to ON/OFF ratio problem by nature. When we limit the ON/OFF ratio of the RRAM devices, the recognition accuracy of the proposed architecture remains almost unchanged. This is because when the architecture compares squared Euclidean distance values, the influence of the OFF state current is offset by each other.

Supplementary Note: SPICE model of RRAM for pulse mode simulations

To simulate the circuit design of the proposed architecture, a SPICE model of our RRAM device has been developed in Verilog-A language. The model can emulate the conductance changes of RRAM in pulse mode properly, including abrupt SET, gradual RESET and device variations. Based on the knowledge that the conductance change of RRAM is relavant to both the applied voltage and its current state, the following equation is used to directly describe the conductance change of RRAM:

$$\frac{dG(t)}{dt} = \gamma v(t) f(v(t), G(t))$$

Where $G(t)$ is the conductance of an RRAM device, which also indicates the current state. $v(t)$ is the voltage across the RRAM device at t, and $f(v(t), G(t))$ is a function of both the voltage and the conductance. γ is a random variable with normal distribution of mean 1 falling in a limited range, it is used to emulate the variations of RRAM during SET/RESET processes. The crucial part of the equation is $f(v(t), G(t))$, which actually defines the relationship between the resistive switching speed and the applied voltage, and the current state itself. In this work, we adopt a simplified method to define the function $f(v(t), G(t))$. The function operates a logical judgement:

$$f(v(t), G(t)) = \begin{cases} c_1 & v(t) \le -V_1 \\ h(G(t)) & -V_1 < v(t) \le -V_2 \\ 0 & -V_2 < v(t) \le V_3 \\ c_2 & v(t) > V_3 \end{cases}$$

Where $-V_1$ and $-V_2$ specify a range of applied voltages that RRAM can show gradual RESET characteristics, and V_3 is the critical voltage that RRAM starts to show abrupt SET. c_1 and c_2 are constants. $h(G(t))$ is a piecewise constant function of the conductance. Besides, some specific restrictions are also defined, for instance, the maximum and minimum conductance, the maximum resistive switching speed, etc. Without any complicated functions, the model is suitable for large scale simulations. In our simulation, the model also exhibits high simulation speed and good convergence, which are very crucial in this work. After calibrating the SPICE model with the experiment data, it turns out that the model can reproduce the characteristics of the fabricated RRAM device in pulse mode properly, as shown in Supplementary Fig. 3 (a) - (b).

It is worth noting that this model can only emulate the RRAM characteristics in pulse mode rather than DC mode. Since RRAM always works in pulse mode in the proposed architecture, the SPICE model above is suitable for this work.

References

1. Wong, H.-S. P. et al. Metal-oxide RRAM. *Proc. IEEE* **100,** 1951–1970 (2012).
2. Chen, B. et al. Highly compact ($4F^2$) and well behaved nano-pillar transistor controlled resistive switching cell for neuromorphic system application. *Sci. Rep.* **4,** 6863-6863 (2014).
3. Akinaga, H. & Shima, H. Resistive random access memory (ReRAM) based on metal oxides. *Proc. IEEE* **98,** 2237-2251 (2010).
4. Yang, J. J., Strukov, D. B. & Stewart, D. R. Memristive devices for computing. *Nature Nanotechnology* **8,** 13-24 (2013).
5. Prezioso, M., Merrikh-Bayat, F., Hoskins, B. D., Adam, G. C., Likharev, K. K. & Strukov, D. B. Training and operation of an integrated neuromorphic network based on metal-oxide memristors. *Nature International Weekly Journal of Science* **521,** 61-64 (2014).
6. Lee, H. Y., Chen, Y. S., Chen, P. S. & Gu, P. Y. Evidence and solution of over-reset problem for HfOx based resistive memory with sub-ns switching speed and high endurance. *IEEE IEDM* (2010).
7. Sheu, S. S. et al. A 4Mb embedded SLC resistive-RAM macro with 7.2ns read-write random-access time and 160ns MLC-access capability. *IEEE ISSCC* (2011).
8. Chen, Z. et al. High-performance HfOx/AlOy-based resistive switching memory cross-point array fabricated by atomic layer deposition. *Nanoscale Research Letters* **149,** 198-205 (2015).
9. Liang, J. & Wong, H. S. P. Cross-point memory array without cell selectors—device characteristics and data storage pattern dependencies. *IEEE Trans. Electron Devices* **57,** 2531-2538 (2010).
10. Yu, S., Gao, B., Fang, Z., Yu, H., Kang, J. & Wong, H. S. P. Stochastic learning in oxide binary synaptic device for neuromorphic computing. *Frontiers in Neuroscience* **7,** 186-186 (2013).
